# Is methotrexate safe for men with an immune-mediated inflammatory disease and an active desire to become a father? Results of a prospective cohort study (iFAME-MTX)

**DOI:** 10.1136/ard-2023-224032

**Published:** 2023-06-01

**Authors:** Luis Fernando Perez-Garcia, Esther Röder, Bouwe P Krijthe, Laura JC Kranenburg-van Koppen, Roxanne van Adrichem, Els Zirkzee, Pieter H Griffioen, Kris Peeters, Marry Lin, Eduard A Struys, Gerrit Jansen, Martijn BA van Doorn, Robert de Jonge, Gert R Dohle, Radboud JEM Dolhain

**Affiliations:** 1 Department of Rheumatology, Erasmus Medical Center, Rotterdam, Netherlands; 2 Department of Rheumatology, Sint Franciscus Vlietland Group, Rotterdam, Netherlands; 3 Department of Rheumatology, IJsselland Hospital, Capelle aan den IJssel, Netherlands; 4 Department of Rheumatology, Maasstad Ziekenhuis, Rotterdam, Netherlands; 5 Department of Clinical Chemistry, Erasmus Medical Center, Rotterdam, Netherlands; 6 Centre for Reproductive Medicine, University of Antwerp, Antwerpen, Belgium; 7 Department of Laboratory Medicine, Amsterdam University Medical Center, Amsterdam, Netherlands; 8 Department of Rheumatology and Clinical Immunology, Amsterdam University Medical Center, location VUmc, Amsterdam, Netherlands; 9 Department of Dermatology, Erasmus Medical Center, Rotterdam, Netherlands; 10 Department of Urology, Erasmus Medical Center, Rotterdam, Netherlands

**Keywords:** Methotrexate, Arthritis, Antirheumatic Agents

## Abstract

**Introduction:**

Current scientific evidence guiding the decision whether men with an active desire to become a father should be treated with methotrexate (MTX) remains controversial. We aimed to prospectively evaluate the testicular toxicity profile of MTX focusing on several markers of male fertility, including semen parameters and sperm DNA fragmentation index (sDFI). As a secondary outcome, we aimed to evaluate whether MTX-polyglutamates can be detected in spermatozoa and seminal plasma and to evaluate the enzymatic activity in spermatozoa of folylpolyglutamate synthetase (FPGS).

**Methods:**

In a prospective cohort study, men ≥18 years who started therapy with MTX were invited to participate (MTX-starters). Participants were instructed to produce two semen samples (a pre-exposure and a post-exposure sample after 13 weeks). Healthy men ≥18 years were invited to participate as controls. Conventional semen analyses, male reproductive endocrine axis and sDFI were compared between groups. FPGS enzymatic activity and MTX-PG1-5 concentrations were determined by mass spectrometry analytical methods.

**Results:**

In total, 20 MTX-starters and 25 controls were included. The pre-exposure and postexposure semen parameters of MTX-starters were not statistically significant different. Compared with healthy controls, the conventional semen parameters and the sDFI of MTX-starters were not statistically significant different. These data were corroborated by the marginal accumulation of MTX-PGs in spermatozoa, consistent with the very low FPGS enzymatic activity associated with the expression of an alternative FPGS splice-variant.

**Discussion:**

Treatment with MTX is not associated with testicular toxicity, consistent with the very low concentration of intracellular MTX-PG. Therefore, therapy with MTX can be safely started or continued in men and with a wish to become a father.

**WHAT IS ALREADY KNOWN ON THIS TOPIC**

- Although methotrexate (MTX) is one of the most frequently prescribed immunosuppressive medication for several immune-mediated inflammatory diseases (IMIDs), the evidence regarding the testicular toxicity profile of MTX is scarce. Ultimately, this has resulted in conflicting recommendations respecting the safety of MTX in men with a wish to have children.

**WHAT THIS STUDY ADDS**

- This is the first prospective study that evaluates the testicular toxicity profile of MTX in men diagnosed with IMIDs. We evaluated the effect of MTX on multiple markers of testicular toxicity and demonstrated that the semen parameters, the male reproductive axis and the sperm DNA fragmentation index were comparable between healthy controls and patients exposed to MTX. Furthermore, our study also demonstrates that MTX can be detected in spermatozoa and seminal fluid, but that the concentration of MTX-polyglutamates, especially in spermatozoa, is very low. Finally, we revealed the mechanistic basis for these latter findings, demonstrating that in spermatozoa, the catalytic activity of the enzyme responsible for the polyglutamylation of MTX, folylpolyglutamate synthetase, is very low.

**HOW THIS STUDY MIGHT AFFECT RESEARCH, PRACTICE OR POLICY**

- Altogether, our data suggest that MTX is not associated with testicular toxicity. Therefore, therapy with MTX can be safely started or continued in men diagnosed with an IMID and with an active wish to become a father.

## Introduction

Methotrexate (MTX) is one of the most frequently prescribed immunosuppressive drugs for the treatment of several immune-mediated inflammatory diseases (IMIDs) such as rheumatoid arthritis (RA), psoriatic arthritis (PsA) and psoriasis. Remarkably, for men with an active desire to become a father, the decision of whether they should stop or continue therapy with MTX before conception remains controversial.

In this regard, the American College of Rheumatology recently updated its recommendations on paternal immunosuppressive exposure before conception.^1^ Concerning MTX, they recommend that MTX can be ‘conditionally continued’ in men planning to father a child. Furthermore, the British Society for Rheumatology guideline on prescribing drugs in pregnancy considers paternal exposure to low-dose MTX (≤25 mg/week) as compatible with pregnancy.^2^ Conversely, other federal agencies and medical associations recommend that MTX should be stopped at least 3–6 months before conception.^3–5^

A fundamental reason for these contradictory recommendations is the scarcity of solid scientific data on the testicular toxicity profile of MTX. In its guideline to evaluate testicular toxicity, the Food and Drug Administration (FDA) considers semen analysis parameters as the main outcomes of interest.^6^ Furthermore, in studies evaluating testicular toxicity, the FDA recommends evaluating at least one baseline and one follow-up semen sample (at the end of the first 13 weeks).

In addition to spermatogenesis, the production of hormones is another important function of the testicles. Therefore, the evaluation of the male reproductive endocrine axis (ie, testosterone, luteinising hormone (LH), follicle stimulating hormone (FSH)) should also be considered an important outcome.^7^

Another novel outcome of interest that reflects the integrity or damage of the sperm DNA and that can be evaluated is the sperm DNA fragmentation index (sDFI).^8^ Sperm DNA integrity is indispensable for the birth of healthy offspring and sperm DNA damage has been strongly associated with male infertility and a higher risk of miscarriages.^9^ Sperm DNA damage can be induced by either the pharmacological exposure itself or the oxidative stress and inflammation states associated with a diagnosis of IMID.^10^

Finally, it is known that the pharmacological efficacy of MTX critically depends on the intracellular bioactivation of MTX to MTX-polyglutamates (MTX-PG). This process is catalysed by the enzyme folylpolyglutamate synthetase (FPGS). Lower FPGS activity and subsequent inefficient polyglutamylation is a well-known phenomenon associated with a rapid efflux of MTX from cells.^11^ MTX-PG have been detected in several human cells such as erythrocytes or peripheral blood mononuclear cells (PBMCs) and low levels of MTX-PG have been associated with increased resistance to therapy.^12 13^ In this regards, recent molecular studies have shown that a significant reduction of FPGS activity is associated with aberrant pre-mRNA splicing of this enzyme, including partial retention of intron 8 (8PR) and a higher ratio of FPGS 8PR over FPGS wild type (WT).^14^ Whether intracellular MTX-PG can be detected in the spermatozoa and whether the spermatozoa have the enzymatic capabilities of forming and retaining intracellular MTX-PG has never been studied before.

To establish whether MTX can be safely used by men diagnosed with an IMID and a wish to conceive, we aimed to prospectively evaluate the testicular toxicity profile of MTX focusing on several markers of male fertility such as semen parameters, the male reproductive endocrine axis and the sDFI. Furthermore, as a secondary outcome, we aimed to evaluate whether MTX-PG can be detected in spermatozoa and seminal plasma and to evaluate the FPGS enzymatic activity in spermatozoa.

## Methods

### Study design and patient selection

The iFAME-MTX study was a prospective cohort study conducted in the Erasmus University Medical Center, Rotterdam, The Netherlands. All participants were aged 18–55 years and were proven fertile (history of a partner’s positive pregnancy test). Three groups of men were included in the study. First, men diagnosed with an IMID (RA, Spondyloarthritis (SpA), PsA, psoriasis) based on the expert opinion of their rheumatologist or dermatologist, who were not exposed to MTX in the last year and were going to start therapy with MTX, were included in the ‘MTX-starters’ group. Second, to evaluate the effect of chronic MTX exposure, men who were exposed to MTX (≥15 mg/week for≥1 year) were included in the ‘MTX-chronic’ group. Third, healthy men were included as ‘healthy controls’.

Men who were not proven fertile, who were exposed to drugs with known testicular toxic side effects (ie, oligospermia) were excluded. Importantly, concomitant therapy with prednisone (≤7.5 mg/day), hydroxychloroquine or tumour necrosis factor alpha (TNFa) inhibitors was allowed (see online supplemental file 1), table 1. Exclusion criteria and table 2 Exclusion criteria, drug exposure).

10.1136/ard-2023-224032.supp1 Supplementary data

**Table 1:** Demographic characteristics

	MTX-naïve Pre-exposure (n=20)	MTX-naïve Post-exposure (n=18)	Healthy controls (n=25)	MTX chronic§ (n=5)	P value
**General information**					
Age years, mean (95% CI)	35.2 (31.4 to 39.1)		34.7 (32.9 to 36.7)	36.6 (32.1 to 41.1)	NS
Smoking, n (%)	4 (20)	4 (20)	6 (24)	1 (20)	NS
BMI %, mean (95% CI)	27.1 (24.8 to 29.2)	26.8 (24.5 to 29.1)	25.5 (24.2 to 26.8)	25.5 (21.4 to 29.6)	NS
Testicular volume, mean (95% CI)	22.9 (21.4 to 24.3)		22.6 (21.3 to 23.8)	22.6 (21.3 to 23.8)	NS
**Inflammatory arthritis**					
Diagnosis:					
RA, n (%)	7 (35.0)	6 (33.3)	-	2 (40.0)	
PsA, n (%)	8 (40.0)	7 (38.9)	-	1 (20.0)	
SpA, n (%)	1 (5.0)	1 (5.6)	-	2 (40.0)	
Psoriasis, n (%)	4 (20.0)	4 (22.2)	-	0 (0.0)	
Age at diagnosis, mean (SD)	27.4 (22.1–32.6)	28.5 (24.5–34.5)	–	30.3 (18.5–42.1)	
Disease duration, mean (SD)	6.9 (2.1–11.8)		^-^	5.6 (-5.5–16.8)	
MTX dose (mg/week), mean (95% CI)	–	16.0 (13.6 to 18.4)	–	18.3 (15.6 to 21.1)	
Prednisone exposure, n (%)	2 (10.0)	6 (33.3)	--	1 (20)	NS
TNFa inhibitor exposure, n (%)	3 (15.0)	5 (27.8)	--	2 (40)	NS
C reactive protein mg/dL, median (IQR)	2.1 (0.6–5.0)	1.4 (1.0–3.2)	0 (0.0–0.9)	1.1 (0–1.8)	* p=0.011 † p=0.008
**Disease activity scores**					
VAS general health mm, mean (95% CI)	42 (19 to 67)	20 (12 to 36)	17.7 (10.5 to 25-5)	20.20 (0.3 to 40.1)	* p=<0.001 ‡ p=0.008
VAS pain mm, mean (IQR)	42 (5.5–75)	14 (4–36)	--	11 (4–34)	
VAS activity mm, mean (IQR)	68 (51–78)	27 (16–49)	--	10 (4–52)	
RA: DAS28, mean (IQR)	2.7 (2.4–2.9)	2.55 (1.34–3.40)	--	2.3 (1.4–2.7)	
PsA: DAPSA, mean (IQR)	24.1 (20.5–33.3)	17.8 (11.2–22.7)	--	1.1 (1.1–1.1)	
Psoriasis: PASI, mean (IQR)	1.9 (1.3–5.1)	1.1 (0.7–3.1)	--	0.8 (0.3–1.2)	

*Statistically significant difference between pre-exposure and healthy controls.

†Statistically significant difference between post-exposure and healthy controls.

‡Statistically significant difference between pre-exposure and post-exposure.

§Presented only for descriptive purposes, no statistical analyses were conducted.

BMI, body mass index; DAPSA, disease activity index for psoriatic arthritis; DAS28, disease activity score 28; MTX, methotrexate; PsA, psoriatic arthritis; RA, rheumatoid arthritis; SpA, spondyloarthritis; TNFa, tumour necrosis factor alpha; VAS, visual analogue scale.

**Table 2:** Conventional semen parameters and sperm morphology

	MTX-naïve Pre-exposure (n=20)	MTX-naïve Post-exposure (n=18)	Healthy controls (n=25)	MTX chronic* (n=5)	P value
**Conventional semen parameters**					
Sperm concentration x10^6^/mL, median (IQR)	57.0 (35.0–90.5)	54.0 (41.0–82.0)	60.0 (37.0–111.0)	37.0 (32.0–59.9)	NS
Progressive motility* %, mean (95% CI)	63.2 (55.4 to 70.9)	60.1 (49.5 to 70.6)	56.9 (51.1 to 62.8)	50.4 (34.8 to 65.9)	NS
Semen volume mL, median (IQR)	2.4 (1.6–3.2)	3.0 (1.5–3.2)	3.0 (2.0–4.0)	2.0 (1.6–2.4)	NS
**Sperm morphology evaluation**					
Normal morphology %, mean (95% CI)	6.4 (4.5 to 8.3)	7.1 (5.6 to 8.4)	6.3 (4.7 to 7.9)	5.9 (2.6 to 9.1)	NS
Teratozoospermia index, mean (95% CI)	1.2 (1.2 to 1.3)	1.3 (1.2 to 1.4)	1.2 (1.2 to 1.3)	1.2 (1.1 to 1.4)	NS
Excess residual cytoplasm, median (IQR)	2.0 (0.7–4.3)	2.0 (1.0–4.5)	2.0 (1.0–4.0)	2.0 (1.0–4.0)	NS
Abnormalities in head (%), mean (95% CI)	92.8 (90.8 to 94.7)	93.0 (91.2 to 94.6)	92.7 (91.0 to 94.3)	92.3 (88.0 to 96.5)	NS
Abnormalities in middle piece (%), mean (95% CI)	19.2 (14.2 to 24.1)	24.5 (18.6 to 30.2)	19.9 (15.3 to 24.5)	22.9 (5.7 to 40.0)	NS
Abnormalities in tail (%), mean (95% CI)	7.1 (3.8 to 10.5)	7.3 (3.7 to 11.1)	6.6 (3.9 to 9.3)	4.6 (1.4 to 7.7)	NS

*Presented only for descriptive purposes, no statistical analyses were conducted.

MTX, methotrexate.

### Study visits

A study visit consisted of three parts. First, the demographic and medical history were obtained from an interview, followed by a concise physical evaluation. Second, a blood sample was obtained. Finally, participants provided a semen sample. Two study visits were required from participants from the MTX-starters group, one before exposure to MTX (pre-exposure) and another one at least 13 weeks after their initial MTX exposure (postexposure). Participants from the MTX-chronic and healthy control groups were just required to complete one study visit.

### Demographic characteristics, medical history and physical evaluation

Participants answered a questionnaire that included questions concerning their demographic characteristics, medical history (including reproductive history) and their current and previous medication exposure.

A physical evaluation was performed to determine height, weight and testicular volume (using an orchidometer). Disease activity was calculated using validated scores (DAS28 for RA, BASDAI for SpA, DAPSA for PsA and PASI for psoriasis).

### Blood sample

Blood from all participants was obtained between 09:00 and 11:00 hours by venipuncture. The concentration of testosterone, LH, FSH, inhibin B, sex hormone binding globulin (SHBG) and C reactive protein were evaluated. Erythrocytes and PBMCs were isolated using the protocol previously described.^12 15^

### Semen sample collection

Semen samples were delivered by masturbation. To ensure a timely analysis of the samples, participants provided the sample in the hospital. All samples were evaluated within 30 min of production. The fresh semen sample was analysed for semen volume, sperm concentration and motility. Slides were prepared for the morphology analysis and sent to a specialised centre (Radboud UMC, Nijmegen The Netherlands).

Thereafter, the semen samples were individually processed to prepare pellets for further analysis. First, samples were processed for future sDFI measurement following the steps previously published.^16^ Second, the semen samples were centrifuged and washed out three times with PBS and spermatozoa were isolated for future MTX-PG concentration, FPGS catalytic activity and mRNA expression measurements. All samples were stored at −80°C.

### Sperm DNA fragmentation index

Assessment of sDFI was performed using the terminal deosynucleotidyl transferase dUTP nick end labeling (TUNEL) assay described by Mitchell *et al*.^17^ In short, the spermatozoa pellets were, after thawing, washed in phosphate buffered saline and incubated in 2 mM dithiothreitol (Sigma-Aldrich, Belgium) for 45 min. After washing, the pellets were incubated in fresh permeabilisation solution (0.1% sodium citrate, 0.1% triton X–100, both Sigma-Aldrich, Belgium) for 5 min at 4°C. The positive control samples were treated with 5 µl of DNase I (Qiagen, Germany) 1500 Kunitz Units for 30 min. The assay was performed using the fluorescein In Situ Cell Death Detection Kit (Roche Diagnostics, Mannheim, Germany) with an Accuri C6 flow cytometer (BD Sciences, Erembodegem, Belgium). For each sample, 5000–10 000 events were recorded at a flow rate of 35 µL/min.

### MTX polyglutamates quantification in erythrocytes, spermatozoa and seminal plasma

#### Procedure EDTA erythrocyte cell pellets

Considering that MTX-PG have never been measured in spermatozoa, we opted to measure MTX-PG in erythrocytes as a control measure for MTX adherence. The erythrocyte cell pellets are measured accordingly to our previously described validated LC-MS/MS method using custom-made stable isotopes of MTX-pg1-7 as internal standards.^12 15^ This method with minor alterations was also used to measure MTX-PG in spermatozoa and seminal fluid.

#### Procedure spermatozoa cell pellets

Spermatozoa cell pellets were kept at −80°C until analysis. To avoid polyglutamate deconjugation activity by γ-glutamyl hydrolase (GGH), semen cells were placed on ice and denatured immediately. A volume of 320 µL perchloric acid (16%) was added to the pellets and mixed immediately to denature. After denaturation samples were supplemented with 200 µL saline and 200 µL internal standards mixture (pg1–pg7). Supernatants were transferred two times into clean tubes after centrifugation (Hettich micro 220R, 10 min, 21 250×g, 5°C) and used for measurements of MTX-PG.

#### Procedure for seminal fluid

Seminal fluid samples were kept at −80°C until analysis. To avoid GGH activity, seminal fluids were shortly thawed on ice before denaturation. A sample volume of 200 µL of previously isolated seminal fluid was used. Because of its high viscosity, samples were denatured with 320 µL acidic methanol (16% perchloric acid in methanol) and mixed immediately to denature the seminal plasma. After denaturation, 200 µL of internal standard mixture (pg1–pg7) was added. Supernatants are transferred two times into clean tubes after centrifugation (Hettich micro 220R, 10 min, 21 250×g, +5°C.) Samples were dried under nitrogen flow (Evaporex EVX-192,+50°C) and dissolved in 720 µL purified water (Millipore) and mixed (IKA MTS4, 10 min, 600 rpm, room temperature (RT)). Samples were filtered (Whatmann mini-uniprep, UN203NPUPP) before being used for MTX-PG measurements.

##### FPGS activity in PBMCs and spermatozoa

FPGS catalytic activity in PBMCs (as positive control) and spermatozoa of healthy controls and MTX-starters were analysed in 10 µg protein extracts and assay mixtures containing 250 mmol/L MTX, and 4 mmol/L ^15^N-labelled L-glutamic acid as subtract concentrations as described by Muller *et al*.^18^ FPGS activity is reported as pmol MTX-PG_2_-^15^N formed/hour/mg protein. In addition, cell extracts of CCRF-CEM and CEM/R30dm leukaemia cells were used a positive and negative controls for FPGS activity, respectively.^19^

##### mRNA expression profiles of folate genes in spermatozoa

An important mechanism of loss of FPGS activity and subsequent inefficient polyglutamylation can occur due to aberrant pre-mRNA splicing of FPGS.^20^ We recently identified a partial retention of FPGS intron 8 (8PR) as a prominent splice variant conferring FPGS dysfunction and decreased MTX polyglutamylation in acute lymphoblastic leukaemia and RA patients.^14^ To evaluate if alterations in FPGS pre-mRNA splicing levels in spermatozoa could explain our findings, an additional experiment was performed. Shortly, RNA was isolated from PBMCs and spermatozoa according to the manufacturer’s protocol (BD Biosciences). RNA (250 ug) was reverse transcribed to cDNA using Moloney Murine Leukaemia Virus (M-MLV; Thermo Fisher Scientific, Waltham, Massachusetts) in a reaction buffer containing random hexamer primers (Roche, Basel, Switzerland), Deoxynucleotide triphosphates (dNTPs) (Roche) and a ribonuclease inhibitor RNasin (Promega, Madison, Wisconsin). Primer sequences (see online supplemental figure 1) and methods used to quantify the levels of FPGS 8PR, FPGS WT are described elsewhere.^14^

### Statistical analysis

Comparisons between the pre-exposure and postexposure MTX-starters groups and healthy controls were tested. Because of the low number of participants included in the MTX-chronic group, we present their data in this article only for descriptive purposes. Categorical variables were presented as number (percentage), and continuous variables are reported as mean±SD, or median±IQR, as appropriate. Continuous variables were compared using a one-way analysis of variance, Tukey post hoc test, paired t-test, Mann-Whitney test and Wilcoxon signed-rank. Categorical variables were compared using χ^2^ tests and Fisher’s exact tests. For linear correlation analysis, we used the Pearson correlation coefficient. The level of significance was set as a two-tailed p≤0.05, and statistical analyses were completed using Stata V.15 (StataCorp-LP).

### Ethics

This study was approved by the ethic review board of the Erasmus University Medical Center in compliance with the Declaration of Helsinki (NL64218·078·18). All participants gave their informed consent.

### Patient and public involvement

Three male patients diagnosed with inflammatory arthritis and who are active members of the research advisory board from the Department of Rheumatology of the Erasmus University Medical Center were involved in the design of the questionnaire and the invitation letter. Together, we carefully assessed the burden on participating patients. We intend to share the results to participating patients and will appropriately disseminate the results.

## Results

Between February 2019 and January 2022, a total of 118 (46 MTX-starters, 49 healthy controls and 23 MTX-chronic) men were invited to participate in the study. In total, 50 men agreed to participate (20 MTX-starters, 25 healthy control and 5 MTX-chronic). Most men who did not participate in the study provided their reasons not to do so (no time for study visits (n=23), unwilling to provide semen samples (n=16), COVID-19 lockdown (n=12), no interest in the topic (n=8), Erasmus University Medical Center being too far away (n=2), reason not provided (n=7). The demographic and clinical characteristics of these men are presented in table 1.

### Conventional semen parameters and sperm morphology

There were no statistically significant differences in the median sperm concentration, semen volume, sperm motility and sperm morphology parameters between MTX-starters and healthy controls. Only one case of oligospermia (<15 million spermatozoa/mL) was observed in an MTX-starter (pre-exposure and postexposure samples, table 2).

### Sperm DNA fragmentation index

The median sDFI was higher in the pre-exposure samples from the MTX-starters (22.0% (IQR 10.7–30.7), but this was not statistically significant different when compared with the postexposure sample (13.1% (IQR 9.5–16.3), p=0·247) and to the healthy controls (13.5% (IQR 8.7–20.2), p=0.257). See table 3.

**Table 3:** Sperm DNA fragmentation index

	MTX-naïve Pre-exposure (n=20)	MTX-naïve Post-exposure (n=18)	Healthy controls (n=25)	MTX chronic* (n=5)	P value
Sperm DNA fragmentation index %, median (IQR)	24.3 (7.1–30.7)	13.1 (9.5–19.9)	13.5 (8.7–20.2)	13.5 (13.3–26.1)	NS

*Presented only for descriptive purposes, no statistical analyses were conducted.

MTX, methotrexate.

### Male reproductive endocrine axis

The median serum concentrations of testosterone, SHBG, LH and FSH were not statistically significant different between the groups. The median serum concentration of inhibin B was statistically significant lower in the pre-exposure (132.5 ng/L (IQR 101.5–179.5)) and postexposure samples of the MTX-starters group (123.0 ng/L (IQR 116.0–179.0)) compared with the healthy controls (189.0 ng/L (170.0–236.0)). See table 4.

**Table 4:** Male reproductive endocrine axis

	MTX-naïve Pre-exposure (n=20)	MTX-naïve Post-exposure (n=18)	Healthy controls (n=25)	MTX chronic‡ (n=5)	P value
Testosterone (nmol/L) median (IQR)	14.6 (11.3–16.2)	13.4 (12.0–15.6)	14.1 (12.8–16.7)	16.3 (16.3–17.1)	NS
SHBG (nmol/L) median (IQR)	26.6 (22.6–34.6)	28.8 (22.5–34.6)	32.6 (25.7–41.9)	35.4 (34.1–38.7)	NS
LH (U/L) median (IQR)	3.1 (2.3–3.9)	2.7 (2.2–3.2)	2.9 (2.2–3.4)	4.10 (4.0–4.1)	NS
FSH (U/L) median (IQR)	4.6 (3.5–5.3)	4.2 (3.2–5.0)	3.7 (3.0–4.5)	4.1 (4.0–4.1)	NS
Inhibin B (ng/L) median (IQR)	132.5 (101.5–179.5)	123.0 (116.0–179.0)	189.0 (170.0–236.0)	92.2 (87.0–203.0)	* p=<0.001 p=<0.001

*Statistically significant difference between pre-exposure and healthy controls.

†Statistically significant difference between post-exposure and healthy controls.

‡Presented only for descriptive purposes, no statistical analyses were conducted.

FSH, follicle stimulating hormone; LH, luteinising hormone; MTX, methotrexate; SHBG, sex hormone binding globulin.

### MTX polyglutamate quantification

**Figure 1:**
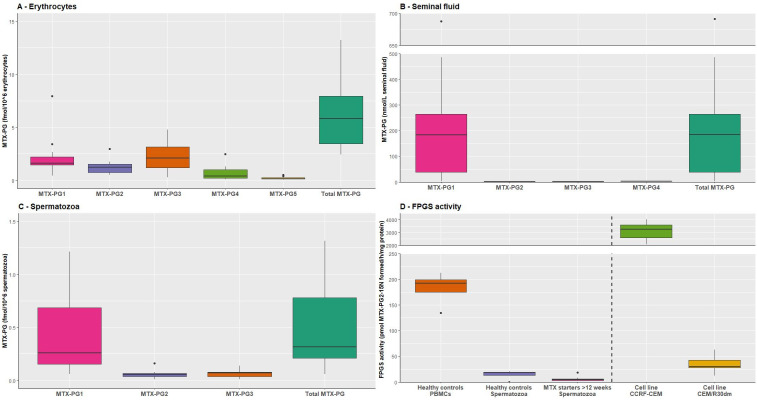
MTX-polyglutamate (PG) accumulation in erythrocytes, seminal fluid and spermatozoa of RA patients and FPGS activity in spermatozoa. Please note that the y axis varies between figures. (A) Individual and total MTX-PG accumulation in erythrocytes of male RA patients (n=17) on MTX therapy for >12 weeks. Data are depicted as fmol MTX-PG/106 erythrocytes and presented in box plots with the sample median and IQR. (B) MTX-PG concentrations (in nmol/L) in seminal fluid of RA patients (n=17) on MTX therapy. (C) Individual and total MTX-PG accumulation in spermatozoa of RA patients (n=17) on MTX therapy. Data are depicted as fmol MTX-PG/106 spermatozoa and presented in box plots with the sample median and IQR. (D) FPGS catalytic activity in spermatozoa of healthy donors (n=4) and RA patients (n=10) for>12 weeks on MTX therapy, and for comparison in PBMCs of healthy individuals (n=4). For comparative and analytical controls, FPGS data are shown for proliferative CCRF-CEM leukaemia cells (n=24) and CEM/R30dm cells (n=15), a subline of CCRF-CEM with 1% residual FPGS activity and with acquired resistance to MTX due to impairment of MTX-PG formation. Data for FPGS catalytic activity are depicted as pmol MTX-PG2-15N formed/hr/mg protein and presented in box plots with the sample median and IQR. FPGS, folylpolyglutamate synthetase; MTX, methotrexate; MTX-PG, methotrexate polyglutamate; RA, rheumatoid arthritis.

MTXpg1–5 were detected in erythrocytes from all participants (figure 1A), consistent with MTX-pg accumulation profiles in erythrocytes of patients under MTX therapy and thus confirming their intake of MTX.^12^ In seminal fluid, mainly MTX-pg1 was detected with a median concentration of 184 nmol/L (IQR 39–265), whereas MTXpg2,3 were barely noticeable (figure 1B). In spermatozoa, mainly MTXpg1 was detected (median 0.26 fmol/10^6^ spermatozoa, IQR 0.16–0.69), whereas MTXpg2,3 levels were at the lower limit of detection (figure 1C). Total MTXpg levels in spermatozoa were approximately 18-fold lower than in erythrocytes (0.32 fmol/10^6^ spermatozoa vs 5.8 fmol/10^6^ erythrocytes, respectively, figure 1A/C).

### FPGS activity in PBMCs and spermatozoa

To determine whether the marginal MTX-pg accumulation is related to FPGS catalytic activity, this enzyme activity was measured in PBMCs and spermatozoa from four healthy controls and 10 MTX-starters (postexposure). FPGS activity (pmol MTX-PG2-^15^N formed/hour/mg protein) was statistically significant higher in PBMCs (183 pmol MTX-PG2-^15^N formed/hour/mg protein) compared with spermatozoa from healthy controls (15 pmol MTX-PG2-^15^N formed/hour/mg protein) and spermatozoa from MTX-starters (5 pmol MTX-PG2-^15^N formed/h/mg protein) (figure 1D). Remarkably, FPGS activity in spermatozoa is even lower than a control cell line (CEM/R30dm) with acquired resistance to MTX due to loss of FPGS activity and impairment of MTX-PG (figure 1D).^19^

### mRNA expression profiles of folate genes in spermatozoa

To explore whether molecular alterations underlie the extremely low FPGS activity in spermatozoa, mRNA expression profiles of FPGS and other folate genes were evaluated in spermatozoa from four healthy controls and six MTX-starters (see online supplemental figure 1). Of note, a relatively high FPGS/8PR and FPGS 8PR/WT ratio was observed in spermatozoa compared with PBMCs (results are not shown).

## Discussion

The iFAME-MTX study is the largest study to date that prospectively evaluated the potential impact of MTX on many important markers of testicular toxicity and to evaluate the potential underlying mechanisms explaining why MTX does not impair sperm quality. It shows that MTX is not associated with conventional semen analysis abnormalities, disturbances in the male reproductive endocrine axis or with increased sperm DNA damage. Furthermore, to the best of our knowledge, our study reports for the first time that the enzyme responsible for intracellular polyglutamylation and hence the bioactivation of MTX, that is, FPGS, has an extremely low activity in spermatozoa. Ultimately resulting in very low concentrations of intracellular MTX-PG in spermatozoa.

Our results provide long-waited answers to important clinical questions. First, studies that evaluated the effect of MTX on semen parameters and/or the male reproductive endocrine axis have resulted in conflicting results.^21^ Most of these studies included a small number of patients, lacked prospectively collected samples, did not have a control group or did not correct for relevant confounders (ie, disease activity or high dose glucocorticoids). Recently, Kazutaka *et al* 22 evaluated the testicular toxicity profile of MTX in a cross-sectional study that included 14 patients mainly diagnosed with Crohn’s disease. Although this was not a prospective study, their findings are similar to ours, as they report that MTX therapy is not associated with abnormalities in semen parameters or the male reproductive endocrine axis.

Second, sperm DNA integrity is essential for producing normal spermatozoa and DNA damage has been associated with male infertility.^23^ Based on the known effects of MTX on DNA synthesis, we were concerned that MTX could result in sperm DNA damage. Reassuringly, we did not find a negative impact of MTX on sDFI. Noteworthy, the median pre-exposure sDFI of 24.3% in MTX-starters may be a reflection of a negative impact of disease activity on spermatogenesis. Although not statistically significant, after exposure to MTX, the sDFI decreased to 13.5%. This may be caused by a reduction of disease activity.

Third, another concern of patients and healthcare professionals is that it was not known whether MTX could be detected in spermatozoa. Therefore, we aimed to measure the concentration of MTXpg, the active forms of MTX in spermatozoa and in seminal fluid. Reassuringly, we detected only MTXpg1 in very low concentrations in spermatozoa and barely detected longer retained MTXpg2,3. Furthermore, the findings of our complementary experiments are reassuring, as we report a very low activity of FPGS in spermatozoa, indicating that MTX polyglutamylation in spermatozoa is limited. Mechanistically, the low FPGS activity in spermatozoa may be associated with a higher ratio of mRNA expression of an alternatively spliced form of the FPGS gene (8PR) over the WT transcript.^18^ Regarding seminal fluid, similar to the recent findings of Grosen *et al*, we detected predominantly MTX-pg1.^22^

The iFAME-MTX study provides a strong scientific basis to consider that MTX is safe for men with an active wish to become a father. Our study showed that exposure to MTX did not result in abnormalities in semen parameters and other male fertility outcomes. Furthermore, although this study was not designed to evaluate the potential teratogenic effect of paternal MTX, three pregnancies that were exposed to paternal MTX were reported by MTX-starters (conception within 1 year after their first study visit). No negative pregnancy outcomes or congenital malformations were reported. This goes in line with our data that shows that MTX is not associated with sperm DNA damage and that polyglutamylation is inefficient in spermatozoa is reassuring. In this regard, the risk of birth defects associated to paternal MTX has been evaluated before in more than 250 men and it was concluded that MTX was not associated with an increased risk of birth defects.^21 24^

Other secondary findings from our study warrant further discussion. Inhibin B is secreted by the Sertoli cells and is considered a marker of Sertoli cell function and spermatogenesis.^25^ Sertoli cells are one of the most important cells necessary for sperm production in men. Comprehensive evaluation of the reproductive axis revealed statistically significant lower serum concentrations of inhibin B in the MTX-starters before exposure to MTX. Lower serum concentrations of inhibin B have also been reported in men diagnosed with ankylosing spondylitis^26^ and systemic lupus erythematosus.^27^ These findings further support the evidence that autoimmunity and inflammation can result in Sertoli cell dysfunction.^22 28 29^ Further research is needed to corroborate these findings.

Furthermore, both findings (higher sDFI and lower inhibin B before exposure to MTX) go in line with the conclusion of our recent study where we reported that inflammatory arthritis might impair male fertility.^30^ Inflammation, especially via mechanisms associated with oxidative stress, was considered as a potential contributor to these findings.^10^ Whether inflammation secondary to IMIDs such as IA results in an increased oxidative stress state in the testicles (or elsewhere) with the potential to disrupt the required homeostasis for normal spermatogenesis remains unknown and warrants further research. Altogether, this may imply that in men with a wish to conceive, treating the disease with immunosuppressive drugs (without known testicular toxicity profiles) while aiming at lower disease activity states, may improve their chances of a successful pregnancy.

Our study has several strengths. It is the first prospective study that included cases and healthy controls and that was specifically designed to evaluate the impact of MTX on several markers of testicular toxicity. Our low loss to follow-up rate maximises the validity of our data. Furthermore, our results were corroborated by the results of our complementary experiments that reported for the first time that polyglutamylation of MTX is very limited in spermatozoa, leading to very low concentrations of the active forms of MTX in seminal fluid and spermatozoa. Our study has important limitations. First, our results are significant and representative for the MTX-starters group and not necessarily of the MTX-chronic group. Second, we only have one semen sample per study visit and not the ideal two (with an average value reported). Thus, the wide known variability of sperm concentration might have influenced our results.

In conclusion, treatment with MTX is not associated with testicular toxicity in men diagnosed with an IMID. It can also be concluded that the concentration of intracellular MTX-PG in seminal fluid and spermatozoa is very low. Therefore, therapy with MTX can be safely started or continued in men diagnosed with an IMID and with an active wish to become a father.

## Data Availability

Data are available upon reasonable request.
