# Supplementary material for: Is methotrexate safe for men with an immune-mediated inflammatory disease and an active desire to become a father? Results of a prospective cohort study (iFAME-MTX)
Source: Ann Rheum Dis. 2023 Jun 1;82(8):1068–75. doi: 10.1136/ard-2023-224032 (PMC10359513; doi:10.1136/ard-2023-224032)
Supplement: Supplementary data [file ard-2023-224032supp001.pdf]

Table 1. Exclusion criteria

MTX-naive and MTX chronic	Healthy controls
• Age above 55 years.<br>• Known infertility (Self-report).<br>• Current use of drugs listed in Table 2.<br>• Current sexually transmitted disease (Self-report).<br>• Current lower urinary tract infection (Self-report).<br>• Active infection with Hepatitis B or C virus (Self-report).<br>• Human immunodeficiency virus (HIV) infection (Self-report).<br>• Vasectomy.<br>• Language barrier.	• Age above 55 years.<br>• Known infertility (Self-report).<br>• Current or past use of Methotrexate.<br>• Current use of any medication.<br>• Current sexually transmitted disease (Self-report).<br>• Current lower urinary tract infection (Self-report).<br>• Active infection with Hepatitis B or C virus (Self-report).<br>• Human immunodeficiency virus (HIV) infection (Self-report).<br>• Vasectomy.<br>• Language barrier.

Table 2. Exclusion criteria – current use of the following drugs (FDA-report of approved drugs that have the potential to impair human spermatogenesis) (29).

Methadone hydrochloride	Nitrofurantoin
Dapsone	Paroxetine
Fluvoxamine maleate	Nifedipine
Colchicine	Cortisone acetate
Dexamethasone	Methylprednisone
Prednisone (>7.5 mg/day)	Sulfasalazine
Triamcinolone hexacetonide	Busulfan
Chlorambucil	Cyclophosphamide
Dabrafenib	Degarelix
Fludarabine	Mercaptopurine
Procarbazine	Triptorelin
Vinblastine	Vinorelbine
Testosterone	
